# Using a combination of fMRI and anterior temporal lobe rTMS to measure intrinsic and induced activation changes across the semantic cognition network

**DOI:** 10.1016/j.neuropsychologia.2014.11.009

**Published:** 2015-09

**Authors:** Richard J. Binney, Matthew A. Lambon Ralph

**Affiliations:** a Neuroscience and Aphasia Research Unit (NARU), School of Psychological Sciences, University of Manchester, UK; b Eleanor M. Saffran Center for Cognitive Neuroscience, Temple University, Philadelphia, PA, USA

**Keywords:** Semantic cognition, TMS, fMRI, Anterior temporal lobe

## Abstract

By developing and applying a method which combines fMRI and rTMS to explore semantic cognition, we identified both intrinsic (related to automatic changes in task/stimulus-related processing) and induced (i.e., associated with the effect of TMS) activation changes in the core, functionally-coupled network elements. Low-frequency rTMS applied to the human anterior temporal lobe (ATL) induced: (a) a local suppression at the site of stimulation; (b) remote suppression in three other ipsilateral semantic regions; and (c) a compensatory up-regulation in the contralateral ATL. Further examination of activity over time revealed that the compensatory changes appear to be a modulation of intrinsic variations that occur within the unperturbed network. As well as providing insights into the dynamic collaboration between core regions, the ability to observe intrinsic and induced changes in vivo may provide an important opportunity to understand the key mechanisms that underpin recovery of function in neurological patient groups.

## Highlights

• First use of combined fMRI and rTMS to explore semantic cognition.
• Intrinsic-automatic and TMS-induced activation changes were identified.
• rTMS induced local suppression and up-regulation in the contralateral ATL.
• Compensatory changes relate to intrinsic variations in the unperturbed network.
• These key mechanisms may underpin recovery of function in neurological patients.

## 1 Introduction

The purpose of this study was to explore the nature of the distributed neural network that underpins semantic cognition. The combination of results from neuropsychology, functional neuroimaging and transcranial magnetic stimulation (TMS), implicates a network of tertiary association regions in multimodal semantic cognition (Binder et al., 2009; Lambon Ralph, 2014), including a bilateral anterior temporal lobe (ATL) representational hub (Lambon Ralph et al., 2010, 2012, 2009; Patterson et al., 2007). TMS can be used to temporarily and focally alter neural activity and thereby investigate the functional necessity of a specific cortical region to a given cognitive process or task. Combining TMS with functional brain imaging (e.g., fMRI), licences examination of how localised changes in neural excitability influence network-wide activity and thereby can be used to reveal causal relationships between brain areas. One particular approach is to administer off-line, low-frequency repetitive TMS (rTMS) to a targeted brain area, with the intention of inducing a ‘virtual lesion’ that lasts for a temporary period during which one can measure brain-wide changes in activity (Knecht et al., 2002; Pascual-Leone et al., 1998; Walsh and Cowey, 2000). This “perturb-and-measure” approach was pioneered in applications to the motor cortex (Lee et al., 2003). Lee and colleagues demonstrated that rTMS applied to the primary motor cortex of neurologically-intact subjects left their motor performance unaffected yet induced a combination of decreased activity in the stimulated area as well as increased motor-related activity within the premotor cortex of the non-stimulated hemisphere. The authors concluded that (i) the up-regulation of activity reflected acute compensatory plasticity which maintained task performance and (ii) such a mechanism might underlie recovery of motor function following unilateral lesions of the motor cortex (e.g., Johansen-Berg et al., 2002). O’Shea et al. (2007) confirmed the compensatory nature of such activation changes by repeating the experiment of Lee and colleagues, and demonstrating that subsequent TMS of the upregulated right premotor cortex disrupted task performance.

Whilst the potential of combined rTMS–fMRI is evident, there have been few attempts to use this approach to investigate cortical networks involved in higher cognitive processes (Andoh and Paus, 2010; Thiel et al., 2006). Whilst an increasing number of studies have used TMS to investigate the neural substrates of cognitive functions such as language and executive control (Andoh et al., 2007; Devlin et al., 2003; Naeser et al., 2005; Pobric et al., 2007), very little is known about how TMS influences neural activity within these specific cortical networks and it remains to be shown whether this influence is either quantitatively or qualitatively different from that demonstrated within sensory-motor cortices (Lee et al., 2003; O’Shea et al., 2007). Moreover, there is much to be learnt regarding if, and how, these networks are capable of generating adaptive, compensatory functional reorganisation. The present study explores these issues by first considering the many methodological challenges associated with combining rTMS and fMRI, and then implementing this approach within the domain of semantic cognition for the first time.

As well as providing important information for neurocognitive models of semantic cognition, our second motivation for using combined rTMS–fMRI was to investigate the potential mechanisms that might underlie the differences between patients with unilateral vs. bilateral ATL damage. Patients with unilateral ATL damage often have mild or minimal semantic impairment in the chronic phase (Bell et al., 2001; Bi et al., 2011; Lambon Ralph et al., 2010, 2012; Seidenberg et al., 2002; Wilkins and Moscovitch, 1978) and certainly much lower levels of impairment than patients with bilateral damage (a pattern that is also observed after ATL resection in non-human primates and monkeys: Brown and Schafer, 1888; Klüver and Bucy, 1939; Klüver and Bucy, 1937). It is possible that this performance difference reflects not only intrinsic computational factors that follow in a bilateral system (Schapiro et al., 2013) but also from the fact that, in aetiologies which cause unilateral ATL damage (e.g., neurosurgery, glioma, and stroke), there can be a period of partial recovery, during which it is possible that the division of labour between the two hemispheres might shift towards the contralateral ATL (Bi et al., 2011; Lambon Ralph et al., 2010, 2012; Schapiro et al., 2013). Combined rTMS–fMRI might provide insights, therefore, about the natural ability of a bilateral representational hub (in the unimpaired brain) to shift the division of labour between the left and right ATLs.

## 2 Materials and methods

### 2.1 General methodological considerations for fMRI–rTMS studies

Given the methodological challenges of combining rTMS and fMRI to explore higher cognition (beyond previous investigations of motor and visual systems), it is important to consider some of the key methodological issues and the limits of what can be expected given the subtle nature of the rTMS effect on cognitive tasks. These are summarised briefly under three subheadings:

(i) *Which aspect of higher cognition should be explored and how* The ideal basis for fMRI–rTMS combinations is cognitive domains where (a) the identity and location of core neural regions are known (from functional neuroimaging and patient lesion studies) and (b) rTMS-behavioural studies of these neural regions have demonstrated consistent behavioural effects. It is also advantageous if identical tasks (to measure the target domain and the ‘control’ paradigms) have been used previously in both fMRI- and rTMS-only studies. As we will show below, the fact that we had explored the core tasks in a previous fMRI experiment, proved to be crucial in the design and analysis of the present fMRI–rTMS study.
(ii) *The experimental design (within or between-subjects)*: unless longitudinal in nature, most fMRI explorations of patient performance (to measure the neural changes following brain damage) have to use a between-subjects design. Most behavioural-only rTMS studies use a within-subjects design so that pre- vs. post-stimulation behaviour can be compared within the same participants (allowing an estimate of the per-subject rTMS effect). Within-subjects designs might be ideal for future rTMS–fMRI experiments but this was not possible in the current study. As shown below, a re-analysis of our previous fMRI-only data highlighted time-related changes during the semantic task. Thus, any comparison of pre- vs. post-rTMS assessment would be confounded with the underlying ‘intrinsic’^1^ changes in neural responsivity. This might not be true for other aspects of higher cognition, of course, or future advances in analysis might be able to model the time effects independently, but the current study does serve as an example of why it is advantageous to have fMRI-only data for the same tasks as those planned for any rTMS–fMRI experiment. The downside of a between-groups design (like cross-sectional patient studies) is that it is not possible to measure the behavioural effect of TMS within the same person and associate this with observed changes in neural activity. In such circumstances, it is seems sensible to limit studies to TMS protocols (site, stimulation and tasks) that have been thoroughly explored and for which the behavioural results have been replicated.
(iii) *Effect sizes and image analysis*: it would appear that, relative to motor or basic sensory domains, both the rTMS behavioural effect and BOLD changes associated with higher cognition are considerably smaller. It is possible that this difference reflects the fact that higher cognition seems to rely on large-scale distributed networks. Thus, stimulation to one element within a large network may have less effect than for domains that rely on concentrated processing within a limited neural region. Secondly, TMS effects are much smaller in scale than the deficits observed in neuropsychological studies. This is true in semantic cognition, for example, where across a series of rTMS experiments (with semantic tasks of varying difficulty) we have rarely obtained changes in accuracy but instead reliable reductions in efficiency (around 10% slowing of decision times: Lambon Ralph et al., 2009; Pobric et al., 2007, 2010a, 2010b, 2009). Consequently, it is possible that the effects on the BOLD responses might also be relatively subtle, in which case, whole-brain image analyses may lack the statistical sensitivity required to detect them (even with a good number of subjects), and an a priori region of interest (ROI) approach might be required.

### 2.2 Design

We conducted exploratory analyses of existing fMRI (no-TMS) data on the same paradigm used in this study (reported in Binney et al., 2010). This explored whether there was sufficient power in the design, if the fMRI run was split into two (a within-subjects design would require TMS-induced effects to be identified by comparing the first and second halves of the data). Importantly and somewhat unexpectedly, these preliminary analyses highlighted significant time-related effects in this fMRI (no-TMS) dataset – particularly when we focussed on the left and right rTMS ROIs (small volume correction performed within a sphere with a 10 mm radius centred over the mean coordinates of left [MNI: −53 4 -32] or right [MNI: 52 2 -28] ATL stimulation reported in the study of Lambon Ralph et al. (2009)). Specifically, these analyses demonstrated a significant down-regulation of semantic activation in the right TMS ROI (*t*(26)=4.11, *z*-score=3.57, *p*_(family-wise error corrected)_=0.02, peak MNI coordinates=53 8 −30, cluster size=68 voxels) implying intrinsic changes in the task-related neural activity over time^2^, which might be related to a similar intra-task reduction of activation and improved reaction times previously described in the context of verb generation (Simpson et al., 2001), see below.

Accordingly, we opted for a between-groups design because it would allow us to look at intrinsic (first vs. second halves of each scanning run within each group) and induced^1^ changes (Control vs. TMS group) independently. Thus, we contrasted the results from Binney et al. (2010) against a new group of fMRI participants who performed the very same tasks but immediately following 1 Hz rTMS to the left lateral ATL (the same neural target used in the previous TMS-only studies: Lambon Ralph et al., 2009; Pobric et al., 2007, 2010a, 2009). As well as contrasting the two groups across the entire fMRI run, we also explored variations over time in both the TMS and no-TMS groups. Inferences were made using both a whole-brain and an ROI-based approach.

### 2.3 Participants

A total of 31 healthy participants took part in the study. All were native English speakers and right-handed, yielding a laterality quotient of at least 75 on the Edinburgh Handedness Inventory (Oldfield, 1971). The control (no TMS) group contained 14 individuals (9 males; age range=19–36 years, mean age=22.1, SD=4.8). Data for this group were originally acquired for analyses that have been reported in a previously published study (Binney et al., 2010). The TMS group was made up of 17 people (9 males; age range=18–46 years, mean age=23.4, SD=7.7) from which we had not previously acquired imaging, TMS or behavioural data. The experiments were reviewed and approved by the local ethics board.

### 2.4 Task and stimuli

A PC running E-Prime software (Psychology Software Tools, Pittsburgh, PA) was used for the presentation of stimuli. A block design was used, with each block lasting 20 s. There were a total of 24 blocks of semantic judgement trials and 24 blocks of number judgement trials. Within each semantic and number judgement block, there were 4 trials. Each trial lasted 5000 ms, comprising a fixation cross presented for 1000 ms followed by the stimuli which were presented for a fixed duration of 4000 ms, in a black, lower-case font on a white background. The participants were asked to respond to the stimuli by pressing one of three designated buttons on a MR-compatible response box.

The semantic judgment task was originally developed to test comprehension in semantic dementia (SD) and other aphasic patient groups (Jefferies and Lambon Ralph, 2006; Jefferies et al., 2009) and has been used in our previous ATL rTMS and fMRI experiments (Binney et al., 2010; Lambon Ralph et al., 2009; Pobric et al., 2007). In this task, the participant was asked to choose which of three choice words was most related to a probe word. Accordingly, each trial contained four written words: a probe word (e.g., rogue), the target choice (e.g., scoundrel), and two unrelated choices (e.g., polka and gasket). The four words within each trial were matched for imageability and word frequency (see Jefferies et al., 2009). The number judgement task (again extracted from our previous ATL rTMS studies) had the same format as the synonym judgment task: a probe number was presented at the top of the screen and underneath three number choices were provided. Participants were required to pick which of the three was closest in value. In the rTMS studies we found that, by using double-digit numbers, the resultant number judgement times were typically equivalent to, if not slightly slower than, decision times for the synonym judgement task. Accordingly, any activation observed for the semantic task when directly contrasted against that of the numerical task could not be due to differences in task difficulty (Pobric et al., 2007).

### 2.5 Target site for TMS

Prior to taking part in the main experimental session, we acquired high-resolution T1-weighted anatomical images (acquisition parameters given in the below section) from each member of the TMS group. On each individual's scan, we identified the cortical site of stimulation as 10 mm posterior to the temporal pole along the lateral surface of the middle temporal gyrus. The mean coordinates of this ATL target in standard stereotactic space (according to the MNI protocol) were [−60, 2, −26]. Inter-subject variability in the coordinates of the stimulated site was minimal, the greatest difference being less than 10 mm from the mean in any one axis. The image coordinates of the scalp/TMS coil position that directly overlaid this cortical target were also recorded. Immediately prior to the experimental session, the structural MR scan was co-registered with the participant's scalp using an Ascension Minibird (www.ascension-tech.com) magnetic tracking system and the MRIreg (www.mricro.com/mrireg.html) software package.

### 2.6 rTMS stimulation parameters

In the line with our previous rTMS experiments, we used the “virtual lesion” method (Walsh and Cowey, 2000) in which a train of low-frequency (1-Hz) rTMS is delivered offline (without a concurrent behavioural task). This form of rTMS modulates the level of cortical excitability at the site of stimulation for a temporary period that extends beyond the duration of the rTMS train itself (Knecht et al., 2002; Pascual-Leone et al., 1998). This ‘refractory’ window offers the opportunity to measure the change in behaviour or neural activity following localised cortical disruption without the complications of simultaneous stimulation or any secondary effects such as muscle contraction, etc.

At the beginning of each TMS session, the individual's hand motor threshold was determined; stimulation was delivered to the ‘hand’ area within the left primary motor cortex and the minimal stimulation intensity required to induce contraction of the relaxed contralateral abductor pollicis brevis muscle (in a minimum of 5 out of 10 trials) was established. rTMS was performed using a MagStim Super Rapid stimulator (The MagStim Company, Whitland, UK) connected to a figure of eight coil (70 mm outer diameter, maximal output of 1.8 T each) and controlled via a PC running Signal software (v2.16, Cambridge Electronic Design, Cambridge, UK). Each participant within the TMS group received 11 min of active 1 Hz rTMS applied to the ATL site (at 120% of motor threshold level) in two blocks of 330 pulses (5.5 min each) with a 30-s inter-train interval (660 pulses in total). Following our previous ATL rTMS protocol (Lambon Ralph et al., 2009), prior to the delivery of the first train of rTMS, coil orientation was adjusted (by rotating around the scalp target, no greater than 45° from the starting orientation where the coil handle is oriented down the length of the middle temporal gyrus) to minimise contraction of facial muscles and thus maximise comfort. Previous rTMS studies, utilising this figure-of-eight coil, have shown that the behavioural effect is invariant to coil orientation (Niyazov et al., 2005), and we have found the same in pilot studies of varying coil orientation over this lateral ATL target.

### 2.7 Transferring the participant from stimulator to scanner

Various measures were taken to minimise the time elapsed between the completion of the rTMS train and the commencement of the functional imaging acquisition. Each participant was familiarised with the scanner environment (including positioning in the scanner, head and leg padding, etc.), the response system and all safety procedures, and was fitted with protective ear plugs, prior to beginning the rTMS train. Stimulation was performed directly outside of the scanner room. Including the time required for preliminary survey scans, an average of 3 min and 50 s (ranging from 3 min 19 s to 6 min 50 s) elapsed between completion of the rTMS and the beginning of the fMRI acquisition.

### 2.8 Imaging acquisition

All imaging was performed on a 3 T Philips Achieva scanner using an 8 element SENSE head coil with a sense factor of 2.5. Functional images were acquired in accordance with the method reported by Embleton et al. (2010; also see below) to reduce the problems associated with imaging the anteroventral and polar temporal cortex. We used a spin-echo echo-planar imaging sequence (SE-EPI fMRI) which included 42 slices covering the whole brain with echo time (TE)=70 ms, time to repetition (TR)=4150 ms, flip angle=90°, 96×96 matrix, reconstructed in-plane resolution 2.5×2.5 mm, slice thickness 3.0 mm. The combination of semantic and control blocks equated to 235 scans. The images were acquired with a single direction *k* space traversal but with a left–right phase encoding direction. In addition, brief (10 volumes for each *k* space traversal) dual direction *k* space traversal SE EPI scans with matching parameters were acquired in order to achieve two sets of images with opposing direction distortions (left–right and right–left). These scans functioned as part of the distortion correction procedure (see below).

In addition, a high resolution T2 weighted turbo spin echo scan with in-plane resolution of 0.94 mm and slice thickness 2.1 mm was obtained as a structural reference to provide a qualitative indication of distortion correction accuracy. Furthermore, high resolution T1-weighted 3D turbo field echo inversion recovery images were acquired (TR≈2000 ms, TE=3.9, TI 1150, flip angle 8°, 256×205 matrix reconstructed to 256×256, reconstructed resolution 0.938×0.938 mm, and slice thickness of 0.9 mm, SENSE factor=2.5), with approximately 270 slices covering the whole brain (variability due to head size). These images were used for identifying the cortical site of stimulation (see above) and also for estimating transforms to warp functional images into standard stereotactic space (see below). The dual direction *k* space images and the anatomical scans were acquired at the end of the scanning session in order to minimise the time elapsed between the rTMS train and the experimental fMRI.

### 2.9 Distortion correction

The spatial remapping correction was computed using a method reported in detail elsewhere (Embleton et al., 2010; Visser et al., 2010a). Briefly, in the first step, each volume of the main functional time-series was registered to the original distorted mean pre-scan volume using a 12‐degrees of freedom affine registration algorithm with Sinc interpolation (FLIRT, FSL, Oxford, UK). Although this initial step was taken primarily as part of the distortion correction procedure, it also functioned to correct the functional EPI volumes for minor motion artefacts. Subsequently, a spatial transformation matrix was calculated from the opposingly-distorted, pre-scan images and then applied to all time points in the functional acquisition. This resulted in a distortion-corrected dataset of 235 volumes maintaining the original temporal spacing and TR of 4150 ms.

### 2.10 FMRI data analysis

All of the following pre-processing steps and analyses were carried out using statistical parametric mapping software (SPM8: Wellcome Trust Centre for Neuroimaging, London, UK) and the general linear model approach. Prior to distortion correction, motion parameters were estimated for each subject by registering each functional EPI to the mean using a rigid body spatial transform and a least squares approach. Following distortion and motion correction (see above section) slice-timing correction was performed using SPM8's Fourier phase shift interpolation and referencing to the middle slice.

Each within-subject data set was then entered into a fixed-effects analysis in which each regressor was modelled as a box car function and subsequently convolved with the canonical haemodynamic response function. The motion parameters were also entered as regressors of no interest. Data were treated with a high pass filter with a cut-off of 128 s. Model estimation used the Restricted Maximum Likelihood approach. In order to assess both the effects of TMS and a potential interaction with time without artificially inflating the degrees of freedom within the random effects analyses, it was required that we used two fixed effects models. The first included two regressors of interest, one modelling the semantic judgment task and the other modelling the numerical judgment task. In this case, contrast images were calculated to assess differences in activations between the semantic task and the control task [semantic judgement-numerical judgement] across the entire scanning run. In the second fixed-effects model, the first 12 blocks of the semantic task were modelled by one regressor and the second 12 by another, and likewise for the numerical task (a total of 4 regressors of interest). Here, contrast images were calculated to provide voxel-wise estimates that represented activity for the semantic vs. the numerical task in the first and second half of the scanning run (semantic 1st-numerical 1st, semantic 2nd-numerical 2nd).

These contrast images were registered to the standard stereotaxic space, according to the Montreal Neurological Institute (MNI), using the DARTEL (diffeomorphic anatomical registration through an exponentiated lie algebra) toolbox (Ashburner, 2007; Ashburner and Friston, 2005), as follows. For each subject, the T1-weighted anatomical image was registered to a mean of the co-aligned motion-corrected (see above section) functional EPI images using a 6 parameter rigid-body transform. SPM8's new unified segmentation was then used to segment these anatomical images into native space tissue components. Subsequently, DARTEL was used to create grey and white matter templates that are representative of the brain size and shape of all the participants plus invertible and smooth deformations (flow fields) for each subject's native space image to this common coordinate space. We then used DARTEL's ‘normalise to MNI space’ option to warp and reslice contrast images into MNI space (resampling to a 1.5×1.5×1.5 mm voxel size). This function estimates an affine transformation mapping between the grey matter group template and a grey matter tissue probability in MNI space, and combines this with the flow fields to produce single deformations mapping from native image space to MNI space. Smoothing is also applied during DARTEL warping and in this case was done so with an 8 mm full-width half-maximum Gaussian filter.

Multi-subject analyses were then carried out on the normalised contrast images using a random-effects model estimation. Group-specific semantic activation was assessed using a one-sample *t*-test on the [semantic judgement-numerical judgement] contrast images taken from a given group. The effect of TMS was assessed by comparing the [semantic judgement-numerical judgement] contrast across the two groups using an independent two-sample *t*-test (assuming unequal variance and independence between the measures). The [semantic 1st-numerical 1st] and [semantic 2nd-numerical 2nd] contrasts were entered into a factorial analysis of variance (ANOVA) model, with the factors group (control vs. tms; assuming unequal variance and independence between levels) and time (1st half vs. 2nd half; assuming equal variance and dependence between levels). This analysis allowed us to explore the possibility of an interaction between group and time and thereby deconfound time effects from the effect of TMS. Note that had we explored the effect of TMS/Group using the factorial model in SPM8, the degrees of freedom would have been artificially inflated (58, compared to 29 in the two-sample *t*-test).

Within whole-brain analyses, inferences were made at the cluster-level, using cluster extent thresholds that were corrected for family-wise error (FWE) using random field theory, as is implemented in SPM8. Small volume corrections (SVCs) were performed exclusively within two a priori ‘TMS’ ROIs (see below) with activations only being considered if they survived or were close to surviving a *p*<0.05 FWE-corrected voxel-height threshold. Further region of interest analyses were conducted using the MarsBar region of interest toolbox (Brett et al., 2002). These ROI analyses included the calculation of a single summary value to represent activation across all voxels within the ROI (median of the parameter estimates).

### 2.11 Region of interest (ROI) construction

As discussed above, we planned to focus our analyses within a set of regions that have been implicated in semantic cognition on the basis of our prior series of behavioural-only rTMS studies and past functional neuroimaging investigations. Two TMS ROIs were defined as spheres (10 mm radius) that were positioned according to: (1) the mean MNI coordinates of the cortical surface target for TMS (lateral ATL) in the present study (see above) and (2) the equivalent coordinates in the right hemisphere. These ROIs were shifted 5 mm along the *x*-axis in the direction of the sagittal midline to ensure that their full extents lay within the cerebrum and did not contain non-brain voxels. The final MNI coordinates of the centre of mass of the left TMS and of the right TMS ROIs were [−55, 2, −26] and [55, 2, −26], respectively.

A further subset of ROIs corresponded to three other left hemisphere regions commonly activated in functional imaging studies of semantic cognition and language (Binder et al., 2009; Binney et al., 2010; Visser et al., 2010b). Their exact locations were defined on the basis of activation by our previous study that used the same semantic and control tasks but without any rTMS (Binney et al., 2010). We used the peaks of activation clusters to define the centre of mass of 3 spherical ROIs with radius of 10 mm. Only activation clusters that survived a conservative extent threshold of *p*<0.05 (corrected for multiple comparisons across the whole brain) were used (see Section 3). Therefore, these ROIs were defined independently of the TMS group data and represented a set of regions that are maximally differentially activated by the semantic task in the control group (and also fall with the regions typically associated with semantic processing: see Binder et al. (2009) and Visser et al. (2010b) for recent large-scale meta-analyses). We reasoned that if there are effects of TMS and/or time, then they would be most likely to occur within this core set of semantic regions. These ROIs corresponded to the left ventrolateral prefrontal cortex (centred on the pars triangularis; MNI coordinates of the centre of mass=[−43, 30, −8]), the left ventral ATL (centred on the fusiform gyrus; [−39, −16, −32]), and the left posterolateral temporal cortex (encompassing the ventral superior and dorsal middle temporal gyri and the superior temporal sulcus). In the case of the posterolateral temporal ROI, however, we used the third most activated voxel in the cluster because the two superordinate peaks were positioned too medially or laterally such that the majority of the sphere would cover non-brain voxels or white matter. The selected peak [−60, −33, 2; peak *Z*-value=3.7] ensured that the ROI lay suitably within grey matter. In addition, the contralateral mirror images of these three ROIs defined an homologous subset of ROIs in the right hemisphere (lateral ATL=[55, 2, −26]; ventral ATL=[39, −16, −32]; posterolateral temporal=[60, −33, 2]; ventrolateral prefrontal=[43, 30, −8]).

Finally, for the MarsBar analyses, each of the ROIs was trimmed using a binary mask generated from grey matter component of the group template (threshold at *p*<0.4, and warped into MNI space via an affine transform, as above). This ensured the exclusion of white matter and extra-cerebral voxels from the ROIs. The TMS ROIs used in the SVCs were left untrimmed.

## 3 Results

### 3.1 Whole-brain multi-subject analyses

#### 3.1.1 Group-specific semantic activation

**Fig. 1:**
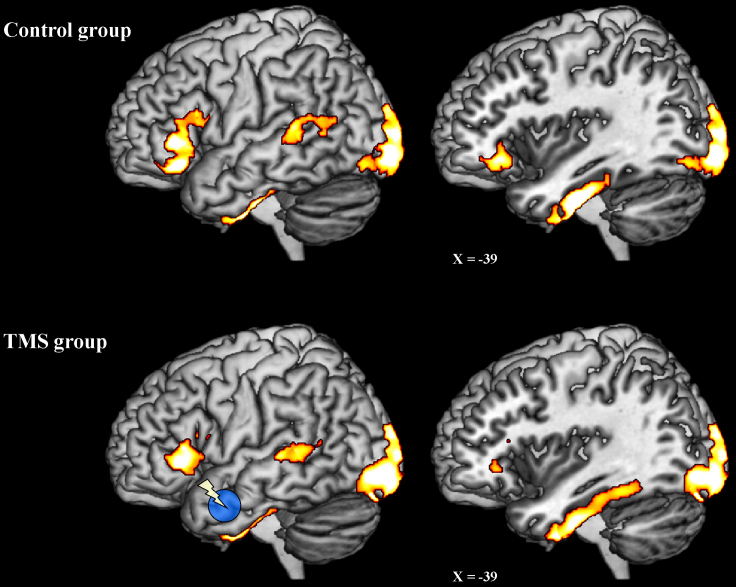
Semantic activation revealed by two independent whole-brain analyses of the control (no-TMS) and TMS groups. Top row: activation revealed by the semantics-numbers contrast in the control (no TMS) group. Bottom row: activation revealed by the semantics-numbers contrast in the TMS group. The activation maps displayed in this figure were subject to a voxel-height threshold of *p*_uncorrected_<0.001, and a *p*<0.05 FWE-corrected cluster-extent threshold (see main text for further details).

First, we examined semantic activation within the control group alone using a one-sample *t*-test (see Section 2). The group statistical image was assessed for cluster-wise significance using a cluster defining threshold of *p*_uncorrected_=0.001, and a *p*_FWE-corrected_<0.05 critical cluster size of 310 voxels (volume=520784 voxels; smoothness [FWHM in mm]=13, 12.4, 8.9; RESELS=1156.6). Table 1 displays the peaks of those clusters that exceeded the critical cluster size. These clusters are also displayed in the top row of in Fig. 1. Similarly, we examined brain-wide semantic activation within the TMS group alone. This statistical image was assessed for cluster-wise significance using a cluster defining threshold of *p*_uncorrected_=0.001, and a *p*_FWE-corrected_<0.05 critical cluster size of 340 voxels (volume=520784 voxels; smoothness [FWHM in mm]=13.2, 12.6, 8.5; RESELS=1167.5). Table 2 lists the peaks of those clusters that exceeded the critical cluster size, while these clusters are also displayed in Fig. 1. As can be seen in Fig. 1, the two groups exhibited very similar semantic activation profiles. There are two important things to note here. First, we have successfully replicated the results of the whole-brain analysis reported in Binney et al. (2010) within an entirely new group of subjects (the post-TMS group). Second, in both groups, the semantic task (relative to the control task) activated regions that are frequently reported in fMRI and PET studies of semantic memory and language (ventral ATL, posterolateral temporal lobe and inferior prefrontal cortex; Binder et al., 2009; Binney et al., 2010; Visser et al., 2010b)) and are also implicated in semantic cognition on the basis of TMS and neuropsychological studies (Corbett et al., 2009; Devlin et al., 2003; Jefferies and Lambon Ralph, 2006; Noonan et al., 2010; Whitney et al., 2011). The ventral ATL activation peaked within the left fusiform gyrus, peaking in its most anterior third but extending over much of its surface. Activation in the left ventrolateral prefrontal cortex, included pars triangularis (BA45) and pars orbitalis (BA47), and possibly also parts of pars opercularis (BA44). Activation was also present within the superior posterolateral temporal lobe. There was also a large cluster of bilateral occipital lobe activation (BA17/18/19) perhaps reflecting greater visual processing required for orthographic over digit stimuli or semantic feedback to early visual areas (Hon et al., 2009).

#### 3.1.2 The effects of TMS and interactions between TMS and Time

Next, we examined how semantic activation differed between the control and TMS groups (see Section 2 for details regarding this random effects analysis). We searched for effects in either direction (tms>control and control>tms), applying a voxel-height threshold of *p*_uncorrected_<0.001, and a *p*_FWE-corrected_<0.05 (430 voxels) cluster extent threshold. We also examined the data with a more liberal *p*_uncorrected_<0.05 (170 voxels) cluster extent threshold. However, these analyses failed to reveal any significant differences in activation across the entire cerebral cortex. We then focused our analyses within the ATL region targeted with TMS and its right hemisphere homologue by applying a small volume correction (SVC) within a 10 mm sphere centred over each of these ROIs (volume=1012 voxels; see Section 2). Making inferences at the voxel-level, we identified activation within the left TMS ROI that was greater within the control group than in the TMS group (MNI coordinates=[−57, 2, −35]; *t*(29*)*=3.71; *z*=3.31; *p*_FWE-corrected_=0.034; SVC). This result is suggestive of a local suppression effect of rTMS. No effects were found in the right TMS ROI.

We also explored the possibility of an interaction between the effects of ATL TMS (Group) and Time (see Section 2 for more details regarding this analysis), given that we had preliminary evidence suggesting that there are intrinsic changes in task-related neural activity that may confound the effects of TMS (See Section 2). Whole brain analyses using a voxel-height threshold of *p*_uncorrected_<0.001, plus either a *p*_FWE-corrected_<0.05 or a *p*_uncorrected_<0.05 cluster extent threshold, failed to detect any such effects. However, SVCs restricting the search volume to within the left or right TMS ROIs, revealed a TMS×Time interaction that approached significance in both the left lateral ATL ([−55, 8, −21]; *F* (1,58)=11.94; *z*=3.08; *p*_FWE-corrected_=0.068; SVC) and the right lateral ATL ([53, 9, −30]; *F*(1,58)=11.38; *z*=3.00; *p*_FWE-corrected_=0.082; SVC). Furthermore, SVCs performed on one-tailed *t*-tests revealed a positive interaction both in the left TMS ROI ([−55, 8, −21]; *t*(58)=3.46; *z*=3.28; *p*_FWE-corrected_=0.034; SVC) and right TMS ROI ([53, 9, −36]; *t*(58)=3.37; z=3.21; *p*_FWE-corrected_=0.042; SVC). The relatively small effects highlighted by these analyses are consistent with the behavioural effect size of rTMS reported in our previous TMS-only studies (see above) which generates a slowing rather than impairment of semantic decisions – and indicates that fMRI exploration of rTMS effects on cognitive tasks may require an a priori ROI-based approach.

#### 3.1.3 Region of interest analyses

The results from the above analyses prompted us to conduct a series of a priori ROI analyses using the Marsbar region of interest toolbox (Brett et al., 2002), in which we extracted values for regional semantic activation within each group and within each half of the scanning run. Plotting these values would allow us to visualise the nature of the interactions within the TMS ROIs (see above). We also performed exploratory *t*-tests to further interrogate these effects.

**Fig. 2:**
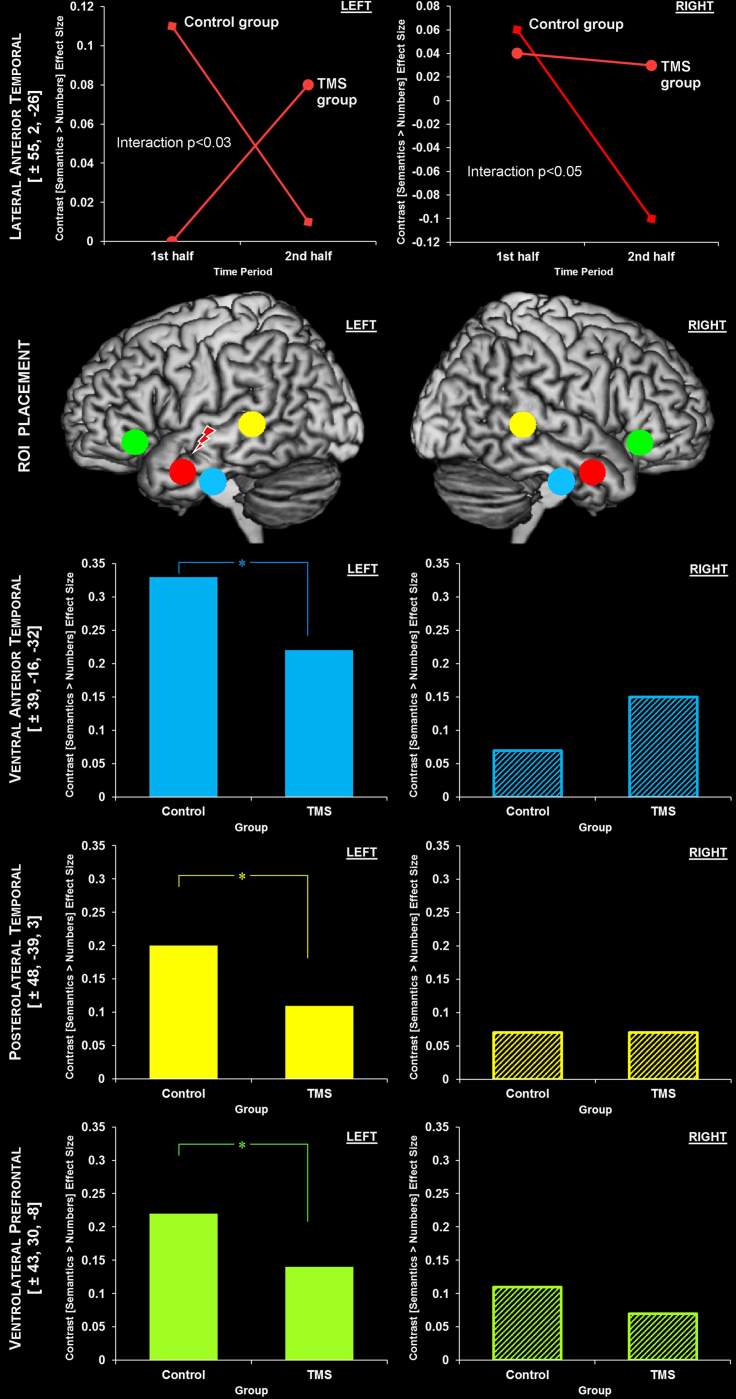
Location and summary of the ROI analyses exploring the effects of TMS and time on semantic activation. Interaction effects are only shown if their associated *p*-value was <0.15. ^⁎^ indicates a between-group (TMS) effect with an associated *p*-value of <0.05.

Furthermore, one of the key aims of the present study was to examine the possibility of ATL rTMS-induced changes across the wider semantic network. Therefore, we extended the analyses beyond the ATL TMS ROIs, to include three other core left hemisphere semantic regions; the ventral ATL, posterolateral temporal cortex and ventrolateral prefrontal cortex (vlPFC; see Fig. 2). These regions are frequently implicated by functional neuroimaging, TMS and patients studies of semantic cognition and language (Binder et al., 2009; Binney et al., 2010; Devlin et al., 2003; Thompson-Schill et al., 1997; Visser et al., 2010b; Wagner et al., 2001). Indeed, they were activated by the semantic task in the present study and in the case of both the Control and TMS groups (with stringent whole-brain FWE-corrected thresholds; see above). We reasoned that if any network wide changes in activation were to arise as a result of ATL rTMS, then they would occur within these maximally differentially activated areas. We also examined their right hemisphere homologues given the possibility that unilateral disruption may invoke contralateral compensatory activity, as suggested by rTMS and patient data from the sensory-motor domain (Johansen-Berg et al., 2002; Lee et al., 2003; O’Shea et al., 2007). However, because there is relatively little evidence of a contribution of these homologous areas to semantic cognition (compared to the left hemisphere regions), either in the healthy or damaged brain, these ROIs were treated with more stringent statistical thresholds (*p*<0.05, Bonferroni corrected). Further details on this set of analyses and ROIs can be found in Section 2, whilst the key findings are summarised in Table 3 and Fig. 2 and shall be elaborated upon in the following paragraphs.

The ROI approach revealed that the left ventral ATL, posterolateral temporal and vlPFC ROIs were significantly more active in the control group than in the TMS group (See Fig. 2 and Table 3). This suggests that, in addition to the local deactivation at the site of stimulation (left lateral ATL; see above), there was remote suppression within the core left hemisphere semantic regions, albeit the activation was not reduced to the level observed during the control task indicating a continued contribution to semantic processing.

A more complex picture emerged as a result of modelling time in the analysis; we observed that in the control group, the left and right lateral ATL were activated by the semantic task, however, unlike the other ROIs (above), activation in both these anterolateral temporal regions greatly decreased over time (see Fig. 2). We confirmed these observations in the control group by using two-sample *t*-tests (assuming equal variance and dependence between levels) to compare activation within these regions in the first and second half of the scanning run; left lateral ATL activation exhibited a trend towards being greater in the 1st half as compared to the 2nd (*t*(26)=1.38, *p*<0.09), whilst this same difference was significant in the right lateral ATL ROI (*t*(26)=2.66, *p*<0.01). Overall, this suggests that under normal neurological circumstances these regions are active during semantic processing but this activity is down-regulated over time. This finding has implications for analyses within future neuroimaging studies of semantic cognition; it demonstrates that in the early phase of a semantic task there is more bilateral activation in anterior temporal regions than the whole brain analyses would suggest, and over time, activation in some of these regions tends to drop away with the effect of producing a more unilateral picture.

Having been subject to low frequency rTMS, however, the left lateral ATL shows a reverse pattern to that observed in the control group and one that would be expected from a local temporary suppression of activation; activity was initially suppressed by direct stimulation but increased as the effects of TMS began to subside in the second half of the scanning run (see Fig. 2). Using two-sample *t*-tests (assuming equal variance and dependence between levels), we sought to confirm this observation by comparing the region's activation in the TMS group across the first and second half of the scanning run; an opposite trend to that in the control group (activation being greater in the 2nd as compared to the 1st half) approached significance (*t*(32)=1.48, *p*=0.07). The ROI analysis also reconfirmed the interaction between time and tms (group) in the left lateral ATL ROI (*t*(58)=1.94, *p*<0.03), that was reported above. Subsequently, therefore, we used further independent two-sample *t*-tests to examine whether the differences between each group within each half were statistically significant (assuming unequal variance and independence between levels). Indeed, in the first half of the experiment, left lateral ATL activation was greater in the control group than in the TMS group (*t*(29)=2.43, *p*=0.011), reconfirming a suppression of local activity following stimulation. In the second half, the activation was numerically greater in the TMS than control group but this difference did not reach significance (*t*(29)=0.83, *p*=0.21). However, one sample *t*-tests confirmed that in the second half, activation within this region approached significance in the case of the TMS group (*t*(16)=1.42, *p*=0.09) but was not significant in the control group (*t*(13)=0.09, *p*=0.46).

A somewhat different pattern emerged in the right lateral ATL. First, the two-sample between-groups *t*-test revealed that this ROI was bordering on being significantly more active in the TMS group than in the control group (*t*(29)=1.46, *p*=0.08) suggesting, like the whole brain analyses, that the local suppression of activity in the left ATL by TMS instigates an up-regulation of activation in the right hemisphere homologue. Moreover, an ANOVA confirmed a significant interaction between the effects of time and tms (group) in this ROI (*t*(58)=1.73, *p*<0.04). Including a factor of time in the analysis revealed that this post-rTMS up-regulation of the right lateral ATL is actually due to an active maintenance of activation (i.e., maintenance of its initial positive level) whereas under normal (non-stimulated) circumstances, activity in this region would normally diminish over time (see above and Fig. 2). We further explored this interaction effect using two-sample *t*-tests (assuming equal variance and dependence between levels); there was no difference between 1st and 2nd time periods in this ROI for the TMS group. Instead, the activation was retained throughout, such that in the second period (when activation had dropped in the control group; see above), the remaining activation was significantly higher in the TMS than control group (*t*(29)=1.65, *p*=0.055).

These results clearly indicate that within this region there are both intrinsic changes in the level of task-related activation over time as well as changes induced by the local effects of TMS itself. Thus, they also reiterate the importance of modelling time in the analyses not only in the TMS group but also in the control group. Without having done so, the analyses would have not detected any difference between the two groups in terms of activation with these regions and the post-TMS compensatory up-regulation in the right hemisphere regions would have been missed; in the standard (non-stimulated situation) the semantic network starts out as a bilateral system and becomes more unilateral over time whereas, following rTMS suppression to the dominant ATL, the semantic network maintains a bilateral distribution – which is consistent with recent neuropsychological and rTMS studies as well as computational models of a bilateral system (Bi et al., 2011; Lambon Ralph et al., 2010, 2009; Schapiro et al., 2013). On a methodological note, this result suggests that the involvement of the right hemisphere can only be revealed if neuroimaging studies/analyses have sufficient power/sensitivity to detect the weaker right hemisphere contribution of these regions (Binder et al., 2009; e.g., in large-scale meta-analyses or studies that include large participant numbers; Price et al., 2005; Visser et al., 2010b) or if intrinsic time-related changes are taken into account.

As noted above, the ATL rTMS produced significant suppression in the other left hemisphere areas within the semantic network. Therefore we performed a final analysis that addressed the question of whether these effects merely reflected a generalised propagation of neural suppression through the cerebral hemisphere or whether they were bound within a functionally-connected network. We addressed this question by performing the exact same analyses as above but within two ‘dummy’ ROIs. The first dummy ROI was a sphere placed over a temporal lobe region (MNI coordinates: [−58 −7 −3]). This region was the same distance from the site of stimulation as the left ventral ATL ROI (the ROI at which the greatest remote TMS-induced suppression was observed) but had not itself been identified as part of the semantic network in the whole-brain analyses (in the middle third of the superior temporal gyrus). Therefore, if there was a generalised spreading of the rTMS suppression effect then this temporal lobe dummy ROI should also show a suppression effect. In contrast, if the suppression is functionally-bound then this region should show no rTMS effect. The second dummy ROI was placed in the left primary motor cortex (MNI coordinates: [−47 −14 45]) which is a classical control site for TMS studies of the visual system and higher cognitive function. One sample *t*-tests revealed that the temporal lobe dummy ROI was significantly active in both the control and TMS group (*p*_uncorrected_=0.05 and *p*_uncorrected_=0.01, respectively) suggesting that it may have some involvement in the semantic task (see Table 3). Critically however, neither dummy ROI was found to exhibit an effect of TMS (Temporal lobe dummy ROI, *p*_uncorrected_=0.71; Primary motor dummy, *p*_uncorrected_=0.26) or a TMS×time interaction effect (Temporal lobe dummy ROI, *P*_uncorrected_=0.52; Primary motor dummy, *P*_uncorrected_=0.30). This result suggests that the remote effects of ATL TMS are indeed functionally bound to the core regions that typically support the semantic task. This analysis, in addition to the pattern of differential effects of TMS on activation of ROIs both within and across hemispheres (i.e., there was neither a set of random effects nor a single global effect; See Fig. 2), also appear to negate the possibility of a non-specific effect of TMS on cortical activity. A sham TMS or control site condition would be required to absolutely rule this out, however.

## 4 Discussion

Contemporary clinical and cognitive neuroscience suggests that semantic cognition is supported by large-scale distributed neural networks (Binder et al., 2009; Lambon Ralph, 2014; Visser et al., 2010b). As such it is important to understand how the various functionally-coupled brain regions interact with one another and how network activity is modulated to cope with variations in computational demands, following changes in the environment, transient disruption or brain damage. In order to investigate these issues we used a combined fMRI–TMS approach in order to measure the effects of localised changes on activity across the network of regions. The transient “refractory” window induced by rTMS also offers the opportunity to model the types of network activity changes that follow brain damage – licensing a better understanding of the mechanisms that support spontaneous, partial recovery of function post brain injury. Methodologically, the current study also delineates the limits of what can currently be achieved and highlights some of the key design choices and required analysis steps in order to detect the activation changes associated with the more subtle nature of rTMS effects on cognitive performance (typically reflecting slowed reactions times rather than the task failure found in neuropsychological studies).

The key findings from the current study were as follows: (1) as hypothesised, rTMS causes a suppression of activity at the targeted brain region (left lateral ATL) which rebounds as the neural effects of stimulation begin to subside; (2) over and above these local effects, TMS induced remote suppression in the other three core, ipsilateral semantic regions, indicating a functionally-bound disruption in the left hemisphere semantic network; and (3) changes in network activity over time revealed both intrinsic and induced plasticity mechanisms; in the control (non-stimulated) group, activity was initially bilaterally distributed but, over time, the right-hemisphere activation diminished resulting in a more unilateral activation profile (an example of *intrinsic* plasticity). Following left ATL suppression, however, this initial right-hemisphere activation was maintained for an extended duration leading to a prolonged bilateral activation pattern (an example of *induced* plasticity). These intriguing results have broad implications for basic and clinical issues – discussed below. We note in advance, however, that the current results are based on a written word, synonym judgement task, which may have biased the activations to the left hemisphere (c.f. Marinkovic et al., 2003). Accordingly, future studies are needed to test if the same patterns are observed for spoken words and nonverbal materials.

### 4.1 Implications for large-scale distributed cognitive networks

The present study has provided insights into the dynamics of large-scale distributed cognitive networks. First, we found that network activity is not stable over time but instead there appear to be *intrinsically*-modulated changes in the distribution of activity; in the unperturbed semantic system, task performance initially received support from a bilateral network but, after some time, network activity was tuned towards the core subset of most active (in this case) left hemisphere regions.

But what is the significance of this down-regulation of activity? First, it may reflect an intrinsic plasticity mechanism that modulates network activity ‘on-line’ in accordance with the precise demands of the current task at any given time and with the ultimate aim of balancing task performance against minimal metabolic expenditure (Attwell and Laughlin, 2001). Initially, there is an overly-generous allocation of processing resources preparing the system for the most challenging of (initially unknown) task demands, followed by a gradual decrease until the most parsimonious and efficient state of activity is reached. In the context of the present task, the right hemisphere elements of the semantic network play a weaker and perhaps secondary role in supporting semantic cognition, and thus provide the best option for down-regulation. A second possibility arises from considering a potentially related effect (Simpson et al., 2001). Simpson et al. found that when participants practiced verb generation repeatedly on the same set of target nouns not only did reaction times reduce but so did the associated regional cerebral blood flow in medial prefrontal cortex and hypothalamus, heart rate and state anxiety ratings. When the participants switched to a new set of words, all these measures reverted back to baseline levels. Whilst these anxiety-related changes in performance might also apply to the current experiment, there are two key differences that need to be taken into account: (a) unlike Simpson et al., we did not repeat the stimuli or trials which were novel on each occasion and (b) the anxiety-related rCBF changes were observed in mPFC and hypothalamus whereas the BOLD changes found in this investigation were observed within the semantic network itself.

These observations were critical to achieving the principal aim of the present study (to measure how localised changes in neural excitability influence activity across the semantic network) because it has become apparent that we needed to know about *intrinsic* changes in activity before we could fully observe and understand those *induced* by the TMS. Specifically, we found that the TMS-induced changes in activity were in fact a modulation of the intrinsic changes observed in the control group. The most striking observation was made within the right lateral anterior temporal lobe; following contralateral TMS, there was an active maintenance of activation (i.e., maintenance of its initial positive level) where, under normal (non-stimulated) circumstances, activity was observed to diminish over time. Thus, in reacting to the induced suppression of activity in its constituent left hemisphere regions (both local and remote), the network appears to retain or up-regulate support from supplementary or subdominant processing resources in order to sustain task performance.

Very similar observations have been reported by studies which have used the combined fMRI–TMS approach to investigate TMS-induced functional reorganisation in the human motor system (see Section 1; Lee et al., 2003; O’Shea et al., 2007) yet, prior to the present paper, there has been little evidence to suggest such responses to TMS perturbation are generalisable to higher cognitive systems. Our novel findings draw a parallel across higher cognitive and motor domains, and appear to confirm that this plasticity principle is a fundamental characteristic of the brain. It might also be the basis of the natural mechanism that supports spontaneous long-term recovery of function following neurological damage (in addition to any performance improvements reflecting strategies and other mechanisms adopted by patients) – a topic discussed in more detail below.

### 4.2 Implications for recovery of function in neurological populations

The typical debate in studies of the neural basis of language recovery following acute brain damage is framed in terms of recovery either reflecting the degree of function in the remaining tissue in the dominant hemisphere or that it depends on a laterality shift to intact contralateral homotopic cortex (Chollet and Weiller, 1994; Price and Crinion, 2005). The latter possibility is often construed as a scenario in which, prior to any damage occurring, the non-dominant, compensatory regions were either dormant or suppressed by transcallosal inhibitory connectivity from dominant regions (with an assumption that the non-dominant hemisphere represents an errant juvenile version of the sophisticated function housed in the dominant hemisphere, which needs to be suppressed to maintain appropriate behaviour when the brain is intact but offers an option for partial function after damage). The present findings suggest an alternative perspective based on different assumptions about how large-scale, bilateral neural networks support cognitive function through dynamic collaboration. Our results indicate that reorganisation of function is constrained to regions which, in the pre-morbid state, had already assumed active roles in the cognitive network. In this context, perhaps the key observation from the current study is that induced changes in the relative contributions of network elements reflect the same processes that underpin intrinsic modulations of the same components. Extending this to the case of neurological damage (i.e., permanent rather than transient induced changes), it would suggest that the same type of intrinsic shifts in divisions of labour across the pre-existing functional network might underpin longer-term recovery processes.

This hypothesis is consistent with functional imaging studies that have demonstrated functional reorganisation associated with recovery following brain lesions or resection for treatment of epilepsy. For example, Bonelli et al. (2012) explored verbal fluency using fMRI before and after anterior temporal resection. In comparison to the typical pattern of left-sided prefrontal activation for this task, Bonelli et al. found enhanced bilateral prefrontal activation in the patients after left ATL resection and that the degree of enhanced right prefrontal activation correlated with post-operative naming abilities. Similarly, neuroimaging studies of language recovery and motor recovery post stroke found not only right hemisphere contributions to function but that these same regions were activated by normal participants (Cappa et al., 1997; Johansen-Berg et al., 2002; Thompson et al., 2000; Weiller et al., 1995), consistent with the notion proposed here that recovery and intrinsic mechanisms for change are one and the same. At least one study has gone further to demonstrate not only that the region implicated in recovery is the same as that used in the undamaged brain, but that there is a key up-regulation of processing in the non-dominant regions. Leff and colleagues found that, in the intact brain, there was an increase in regional cerebral blood flow (rCBF) in the superior temporal gyrus (STG) bilaterally when spoken words were presented at increasing speech rates (Leff et al., 2002). Following infarction of the dominant left STG, the same analysis found not only that the same right STG region supported the patients’ recovered receptive language skills but that there was up-regulation within this area. This change was not simply a global increase in rCBF but rather reflected up-regulation in its response characteristic (the rate of rCBF change as a function of speech rate had significantly increased). The same idea can be observed in computational models of language and recovery of function (Welbourne and Lambon Ralph, 2007; Ueno et al., 2011), where there are divisions of labour across contributing elements within the intact distributed network which can change to compensate for damage. In doing so the compensatory regions do not simply work harder but they exaggerate previous functional characteristics.

### 4.3 Implications for understanding the effects of unilateral vs. bilateral ATL damage

As noted in the Introduction, the combined use of rTMS and fMRI might also provide new insights about the striking performance differences found in patients with unilateral vs. bilateral ATL damage. Typically patients with unilateral damage have mild to minimal semantic impairment in the chronic stage (Bell et al., 2001; Bi et al., 2011; Lambon Ralph et al., 2010, 2012; Seidenberg et al., 2002; Wilkins and Moscovitch, 1978) and certainly much lower levels of impairment than patients with bilateral damage (a pattern that is also observed after ATL resection in non-human primates and monkeys: Brown and Schafer, 1888; Klüver and Bucy, 1939; Klüver and Bucy, 1937). The current results suggest that even written-word semantic tasks engage a bilateral ATL system initially and that this minor right ATL involvement could provide a basis for at least part of the relatively good performance observed in patients with unilateral ATL damage. This might be for two reasons. First, the intrinsic down-regulation of the bilateral to left-lateralised activations might imply that even the meaning of written words is supported bilaterally in the intact brain (but is down-regulated to a unilateral pattern once task performance is established). Thus the (partially utilised) contribution from the right ATL could support good performance after left ATL damage. Secondly, the TMS-induced changes suggest that the right ATL contribution can be maintained when necessary in order to minimise the effects of left ATL damage (analogous to the results obtained in the motor domain: cf. Lee et al., 2003; O’Shea et al., 2007). Extrapolating to unilateral patients, it seems possible that an enhanced version of this compensatory mechanism might support recovery of function post-damage. In the case of bilateral damage, presumably performance is poor because (a) both ATL regions, which contribute to semantic representation, have been compromised and (b) function cannot be upregulated by an intact ATL region as is the case with unilateral damage. Some of these hypotheses have been tested recently in a computationally-implemented bilateral model of semantic representation (Schapiro et al., 2013). Schapiro et al. found that, with regards to the first factor, a bilateral system is intrinsically more robust to the effects of unilateral vs. bilateral damage even when the total amount of damage is held constant (see Schapiro et al. 2013, for a detailed analyses and further explanation of the underpinning factors) and, with regards to the second recovery factor, that this unilateral vs. bilateral difference was amplified when the model was allowed to recover function partially through post-damage connection-weight changes (analogous to plasticity-related changes).

## Figures and Tables

**Table 1:** Significant activation clusters (*p*<0.05, FWE-corrected) for the Control Group, revealed by the whole-brain analysis of semantic>numerical task.

**Brain region**	**Cluster extent**	**Peak*****z*****-value**	**MNI coordinates**		
***X***	***Y***	***Z***
Left FG/ITG	1596	5.42	−39	−16	−32
	4.95	−36	−9	−39
	3.36	−41	−31	−24

Left ventrolateral prefrontal cortex	1679	4.66	−43	30	−8
	4.64	−49	32	1
	3.98	−49	17	19

Left posterolateral temporal lobe	1021	4.03	−48	−39	3
	4.02	−68	−39	11
	3.70	−60	−33	2

Bilateral occipital cortex	7322	5.38	−9	−91	−3
	5.11	20	−88	−5
	5.04	−26	−94	−9

Cluster-defining/voxel-height threshold of *p*<0.001, uncorrected; FG=fusiform gyrus; ITG=inferior temporal gyrus.

**Table 2:** Significant activation clusters (*p*<0.05, FWE-corrected) for the TMS Group, revealed by the whole-brain analysis of semantic>numerical task.

**Brain region**	**Cluster extent**	**Peak*****z*****-value**	**MNI coordinates**		
***X***	***Y***	***Z***
Left FG/ITG	1501	5.00	−34	−3	−44
	4.89	−40	−12	−36
	3.92	−41	−40	−17

Left ventrolateral prefrontal cortex	1319	5.09	−48	26	10
	4.87	−33	35	−3
	4.8	−48	30	0

Left posterolateral temporal lobe	1067	4.51	−51	−40	5
	4.17	−66	−31	5
	4.03	−60	−39	3

Bilateral occipital cortex	12250	5.99	−11	−90	−6
	5.58	−39	−91	−9
	5.23	14	−81	−11

**C**luster-defining/voxel-height threshold of *p*<0.001, uncorrected; FG=fusiform gyrus; ITG=inferior temporal gyrus.

**Table 3:** Summary of the key findings from the ROI analyses exploring the effects of TMS and time on semantic activation.

**Region of interest**	**Highest-order TMS effect**	**Exploratory*****t*****-tests (for interaction effect only)**
Left latATL	Time×TMS Interaction	• 1st half, Control>TMS; *t*=2.43, *p*=0.01 • 2nd half, TMS>Control; *t*=0.83, *p*=0.21 • Control, 1st half>2nd half; *t*=1.38, *p*=0.09 • TMS, 2nd half>1st half; *t*=1.48, *p*=0.07
	*t*=1.94, *p*=0.03
Left vATL	TMS (Control>TMS)	
	*t*=2.04, *p*=0.03	
Left pTL	TMS (Control>TMS)	
	*t*=2.02, *p*=0.03	
Left vlPFC	TMS (Control>TMS)	
	*t*=1.93, *p*=0.03	
Right latATL	Time×TMS Interaction	• 2nd half, TMS>Control; *t*=1.65, *p*=0.05 • Control, 1st half>2nd half; *t*=2.66, *p*=0.01
	*t*=1.73, *p*=0.04
Right vATL	N.S.	
Right pTL	N.S.	
Right vlPFC	N.S.	

Only the highest-order TMS effect with a *p*<0.05 is listed. latATL=lateral anterior temporal lobe; vATL=ventral anterior temporal lobe; pTL=posterolateral temporal lobe; and vlPFC=ventrolateral prefrontal cortex. N.S.=not significant.

## Footnotes

1 By ‘intrinsic’ we mean automatic changes in neural responses that reflect task/stimulus processing and not those associated with the effect of rTMS. For the latter, we will use the shorthand of ‘induced changes’.

2 Two-sample *t*-test (assuming equal variance and dependence between levels) examining differences in semantic activation [semantic judgement-numerical judgement] between the first and second halves of the scanning run. See below for further details regarding data pre-processing and statistical modelling.
